# Galectin‐9 and myeloid‐derived suppressor cell as prognostic indicators for chronic lymphocytic leukemia

**DOI:** 10.1002/iid3.853

**Published:** 2023-05-08

**Authors:** Xierenguli Alimu, Juan Zhang, Nannan Pang, Rui Zhang, Rong Chen, Xuejiao Zeng, Shabaaiti Tudahong, Gang Chen, Maliya Muhashi, Fang Zhao, Jianbing Ding, Jianhua Qu

**Affiliations:** ^1^ Center of Hematology The First Affiliated Hospital of Xinjiang Medical University Urumqi Xinjiang China; ^2^ Senior Department of Hematology The Fifth Medical Center of PLA General Hospital Beijing China; ^3^ CAS Key Lab of Bio‐Medical Diagnostics, Suzhou Institute of Biomedical Engineering and Technology Chinese Academy of Sciences Suzhou China; ^4^ Hematology Institute of Xinjiang Uygur Autonomous Region Urumqi Xinjiang China; ^5^ Department of Immunology, School of Basic Medical Sciences Xinjiang Medical University Urumqi Xinjiang China

**Keywords:** chronic lymphocytic leukemia, Galectin‐9, MDSCs, Tim‐3

## Abstract

**Background:**

Galectin‐9 and myeloid‐derived suppressor cells (MDSCs) have an important role in tumors, but their clinical values in chronic lymphocytic leukemia (CLL) have not been fully elucidated. This study aimed to analyze the prognosis values of Galectin‐9 and MDSCs in CLL.

**Methods:**

The concentrations of Galectin‐9, argininase‐1, and inducible nitric oxide synthase in serum were detected by enzyme‐linked immune sorbent assay. The expression of Tim‐3 protein in peripheral blood mononuclear cell was detected by Western blot. Flow cytometry was used to analyze the percentages of Tim‐3 on T‐cells (CD3^+^T, CD4^+^T, and CD8^+^T cells) and MDSCs.

**Results:**

Our results showed that Galectin‐9 and MDSCs significantly increased in CLL patients and were closely related to the disease progression. Patient's receiver operating characteristic, progression‐free survival, and Cox regression analysis showed that Galectin9 and MDSCs were poor prognostic factors of CLL.

**Conclusion:**

Galectin‐9 and MDSCs were associated with clinical progression and could be important prognostic indicators for CLL.

## Graphical abstract

Galectin‐9 and myeloid‐derived suppressor cells were associated with clinical progression and could be important prognostic indicators for chronic lymphocytic leukemia

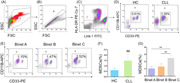

## 1 INTRODUCTION

Chronic lymphocytic leukemia (CLL) is a slow‐developing hematological malignant tumor characterized by clonal proliferation and aggregation of CD19^+^CD5^+^ B lymphocytes in organs such as peripheral blood, bone marrow, lymph nodes, and spleens.^1^ The natural history of CLL is variable with overall survival ranging from 2 to 20 years.^2^ Most patients survive for many years without symptoms or specific treatment, while some patients progress rapidly at the early stage, having worse outcomes.^3^ Therefore, it is very important to evaluate the prognosis of CLL patients accurately.

Our previous study showed that serum Galectin‐9, Tim‐3^+^Th1 cells, and Tim‐3^+^Treg cells are significantly elevated in patients with CLL. Also, the upregulated Galectin‐9/Tim‐3 pathway led to the imbalance of CD4^+^T cell subsets.^4^ As a member of the galectin family, Galectin‐9 is widely expressed in different kinds of immune cells and human tissues; Galectin‐9 can guide tumor cells to escape from immune surveillance and promote tumor development.^5^, ^6^, ^7^ As the receptor of Galectin‐9, Tim‐3 is a newly discovered immune checkpoint molecule mainly expressed on the surfaces of various immune cells.^8^ The Galectin‐9/Tim‐3 signaling pathway has an important regulatory role in many tumors.^9^, ^10^, ^11^ Our previous in vitro experiments showed that blocking the Galectin‐9/Tim‐3 signaling pathway can partially reverse the imbalance of T cell subsets but could not completely control the CLL development.^4^

Myeloid‐derived suppressor cells (MDSCs) increase in multiple tumors such as breast and thyroid cancer and correlate with low survival rates and poor prognosis.^12^, ^13^, ^14^ Under pathological conditions, the differentiation of MDSCs is blocked, and they tend to accumulate. The essential amino acids of T‐cells are depleted by high levels of argininase‐1 (Arg‐1) and inducible nitric oxide synthase (iNOS) produced by MDSCs, which, in turn, inhibits the activation of T cells. This enables tumor cells to escape from immune surveillance and attack, promoting their survival and proliferation in the body.^15^, ^16^ Although many prognostic indicators of clinical and molecular parameters have been established in CLL, they are far from enough. Therefore, searching for new prognostic markers to guide the clinical management of CLL patients is required. The prognostic value of Galectin‐9 and MDSCs in CLL has not been fully understood. Therefore, in this study, we detected the concentrations of Galectin‐9, Arg‐1, and iNOS in serum, the frequency of Tim‐3 on T cells, and MDSCs in peripheral blood. In addition, the prognostic value of Galectin‐9 and MDSCs in CLL patients was evaluated, and the correlation between them was preliminarily explored. Our results may provide a theoretical basis for Galectin‐9 and MDSCs to become a new potential therapeutic target of CLL.

## 2 MATERIALS AND METHODS

### 2.1 Patients

A total of 53 newly diagnosed CLL patients who visited the Department of Hematology of The First Affiliated Hospital of Xinjiang Medical University between January 2020 and October 2022 were included in the experimental group (CLL group). The diagnosis of CLL patients was based on previous literature.^1^ No patient had previously been prescribed either chemotherapy and/or immunotherapy. In addition, 20 healthy controls, who underwent a physical examination at our hospital within the same time period, were included in the healthy control group (HC group). This study was approved by the Ethics Committee of The First Affiliated Hospital of Xinjiang Medical University (20180223‐78). All subjects signed the informed consent form before the start of the study.

### 2.2 Enzyme‐linked immune sorbent assay

The peripheral blood of CLL patients and healthy controls were collected into EDTA collection tubes and centrifuged at 1500 rpm for 5 min. The serum samples were stored at −80°C, and before analyzing, samples were thawed at room temperature and vortexed to ensure a well‐mixed sample. Galectin‐9, Arg‐1, iNOS levels were detected by the corresponding enzyme‐linked immune sorbent assay (ELISA) kits which were purchased from Thermo Fisher. The experimental steps were carried out in strict accordance with the instructions. Within 10 min, the absorbance of each well was measured on a microplate reader (Thermo Fisher) at 450 nm with zero adjustments of the blank control well.

### 2.3 Flow cytometry

Fresh (within 24 h) peripheral blood (2 mL) was collected from CLL patients and healthy controls into heparin‐coated blood collection tubes. Blood samples were well shaken to ensure that the blood was in full contact with heparin to prevent coagulation. The following monoclonal antibodies were used in this study: PE‐labeled anti‐Tim‐3, APC‐labeled anti‐CD3, PerCP‐labeled anti‐CD4, and FITC‐labeled anti‐CD8 for the detection of Tim‐3 expression on the T‐cells. Meanwhile, we set the isotype controls. FITC‐labeled anti‐Lin, PE‐Cy7‐labeled anti‐HLA‐DR, APC‐labeled anti‐CD11b PE‐labeled anti‐CD33 for the detection of MDSCs. Each peripheral blood sample was incubated with 50 μL of the staining cocktail for 15 min in the dark, followed by the addition of 1000 μL of red blood cell lysis buffer. Samples were vortexed well, incubated for 5 min in the dark, and centrifuged at 1500 rpm for 5 min. After decanting the supernatant, the cell pellet was resuspended in phosphate‐buffered saline (PBS, 2 mL) to stop red blood cell lysis, and samples were centrifuged for 5 min at 1500 rpm and washed with 500 μL of PBS. At least 10,000 cells of each sample were analyzed by fluorescence‐activated cell sorting (Canto II). All antibodies and reagents were purchased from BD Biosciences. Analyses were performed using a BD LSRII flow cytometer (BD Biosciences) and Flowjo software (TreeStar).

### 2.4 Western blot analysis

Fresh peripheral blood (5 mL) was collected from CLL patients and healthy controls. Peripheral blood mononuclear cell (PBMC) was isolated using a lymphocyte separation solution (TBD Science). Peripheral blood lymphocytes were lysed in radioimmunoprecipitation assay buffer with protease; phosphatase inhibitors, as well as phenylmethanesulfonylfluoride and the supernatant, were collected after centrifugation. The protein concentration was determined by the bicinchoninic acid method. Sodium dodecyl sulfate (SDS) loading buffer was added to denature the proteins (all the reagents for protein extraction were purchased from Solarbio Company). Proteins were isolated by 12% SDS‐polyacrylamide gel electrophoresis and transferred to the polyvinylidene fluoride (PVDF) membrane (300 mA, 90 min). PVDF membrane was blocked for 1 h by 5% skimmed milk and then incubated with a polyclonal rabbit anti‐Tim‐3 (Proteintech Group) overnight at 4°C. Goat anti‐rabbit (Solarbio) horseradish peroxidase‐conjugated secondary antibody was incubated at room temperature for 1 h. β‐Actin (Cell Signaling Technology) in equal quantities was used as an internal reference. The enhanced chemiluminescence (Biogot Technology) method evaluated the protein expression level.

### 2.5 Statistical analysis

SPSS 26.0 software was used for all data analyses. Measured data were expressed as mean ± SD; the median ± quartile was described as M (P25, P75). For normally distributed data, the *t*‐test was adopted to compare two groups, and one‐way analysis of variance was used to compare data between multiple groups. Fisher's least significant difference was employed for pairwise comparisons. For nonnormally distributed data, the rank‐sum test was used for comparisons. Count data were expressed as the frequency and rate, and group comparisons were performed using the *χ* ^2^ test. Progression‐free survival (PFS) was defined as the interval between the time of follow‐up and the occurrence of disease progression or death from any cause. The Kaplan–Meier method and the log‐rank test were used to assess PFS of CLL patients in different prognostic groups. Univariate and multivariate Cox regression were performed to determine important prognostic factors for CLL patients. Pearson's correlation was employed to detect an association between Galectin‐9 and MDSCs. *p* < .05 was considered a statistical significance.

## 3 RESULTS

### 3.1 Baseline clinical characteristics of patients

There were 53 CLL patients, 32 male and 21 female (the male‐to‐female ratio: 1.52:1), with a median age of 60 (40–85) years. According to the Binet staging system, CLL patients were divided into three groups, including the Binet A stage group (*n* = 14), Binet B stage group (*n* = 28), and Binet C stage group (*n* = 11). In the HC group, 13 patients were male and 7 were female (the male‐to‐female ratio: 1.86:1), with a median age of 61 (55–72) years. No significant differences were observed in gender and age between patients in the CLL group and HC group (all *p* > .05). However, peripheral blood parameters significantly differed between patients in the CLL group and HC group (*p*< .05; Table 1).

**Table 1:** General patient data of the CLL group and HC group ($\bar{x}\pm s$).

Index	HC group (*n* = 20)	CLL group (*n* = 53)	*t*	*p*
Age	61.35 ± 4.25	60.26 ± 10.92	0.43	.67
While blood cell × 10^9^	5.14 ± 0.39	55.14 ± 68.76	3.24	<.01
Lymphocyte × 10^9^	2.06 ± 0.18	39.21 ± 40.31	4.11	<.01
Lymphocyte（%）	30.69 ± 2.45	75.35 ± 16.30	12.15	<.01
Hemoglobin (g/L)	135.50 ± 4.63	124.87 ± 19.72	2.38	<.05
Platelets × 10^9^	246.2 ± 16.38	163.04 ± 68.77	5.33	<.01

Abbreviations: CLL, chronic lymphocytic leukemia; HC, healthy control.

### 3.2 Soluble Galectin‐9 increased in CLL patients

**Figure 1:**
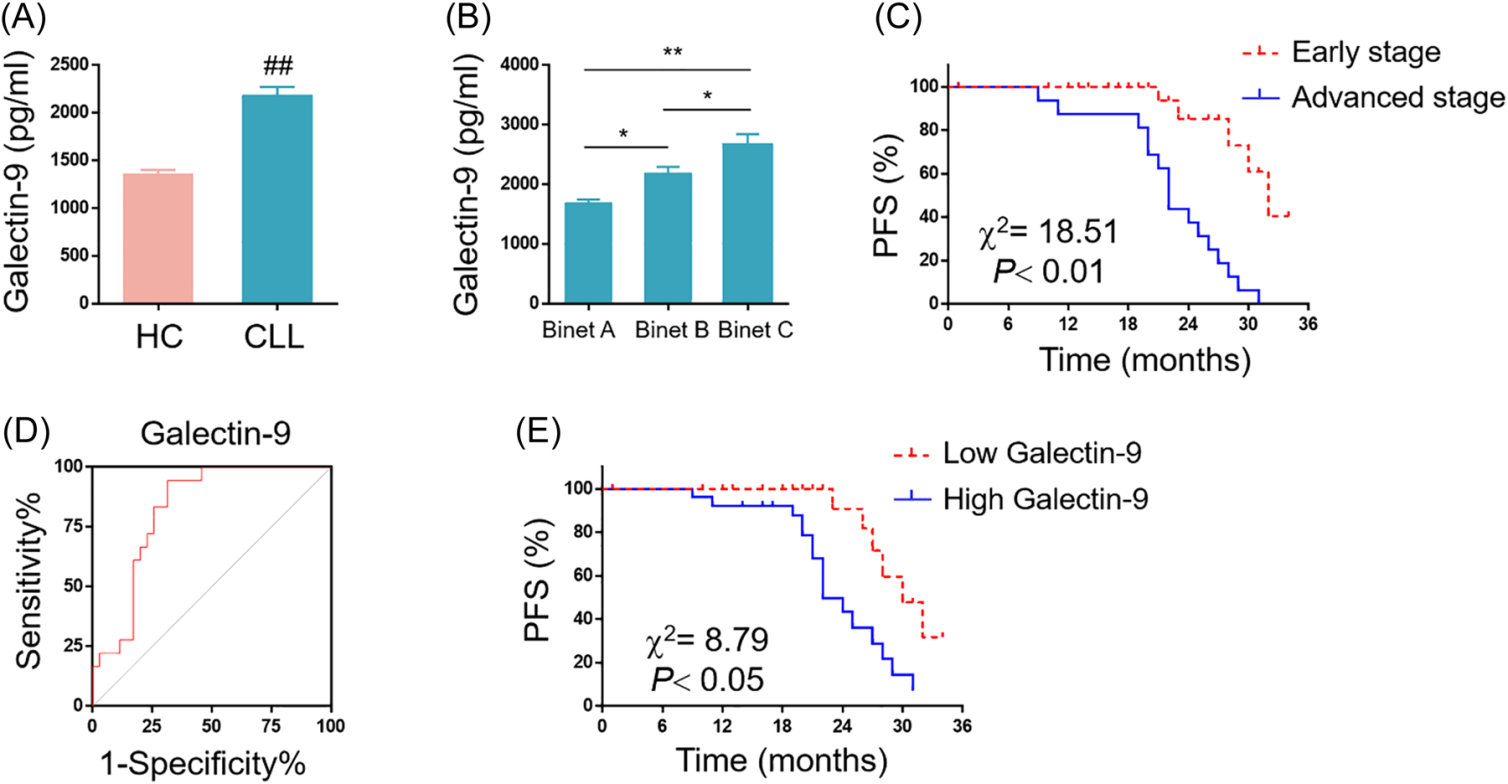
The changes of Galectin‐9 in CLL patients. (A) Serum levels of Galectin‐9 in CLL and HC detected with ELISA. (B) Levels of Galectin‐9 in CLL patients with different Binet stages. (C) The PFS rate of the early and advanced stage in CLL patients (*χ* ^2^ = 18.51, *p* < .01). (D) ROC curve of Galectin‐9 based on the CLL patients of early stages and advanced stages (AUC = 0.81; 95% CI = 0.712–0.933; *p* < 0.05; sensitivity = 94.44%; specificity = 68.57%). (E) Comparison of PFS for different Galectin‐9 levels (*χ* ^2^ = 8.79, *p* < 0.05). Compared with the HC group: ^##^ *p* < .01. *p < .05, **p < .01. AUC, areas under the curve; CI, confidence interval; CLL, chronic lymphocytic leukemia; ELISA, enzyme‐linked immune sorbent assay; HC, healthy control; PFS, progression‐free survival; ROC, receiver operating characteristic.

Our results showed that Galectin‐9 in the peripheral blood serum of the CLL group was maintained at a higher level, as examined by ELISA, relative to the HC group (*p*< .01; Figure 1A). The concentration of Galectin‐9 was significantly different among the three Binet stage groups; it also gradually increased at later stages (*p*< .05, *p*< .01; Figure 1B).

In further analyses, patients were classified as early stage (asymptomatic Binet A and Binet B patients) (*n* = 35) or advanced stage (symptomatic Binet A, Binet B as well as Binet C patients) (*n* = 18) based on whether they had clinical symptoms. The Kaplan–Meier method was used to analyze the PFS rate of the early stage and advanced stage. The result showed that CLL patients in advanced stage presented with a poor PFS (*χ* ^2^ = 18.51, *p* < .01), compared to the early stage (Figure 1C). Receiver operating characteristic curves (ROCs) and defined areas under the curves (AUCs) were generated based on the concentration of Galectin‐9 in the CLL patients of early stage and advanced stage for evaluating the prognostic value of soluble Galectin‐9. The sensitivity for Galectin‐9 was 94.44% and the specificity was 68.57%. Youden index was 2038 pg/mL (AUC = 0.82; 95% confidence interval [CI]: 0.712–0.933; *p* < .05; Figure 1D). CLL patients were divided into high Galectin‐9 group (≥2038 pg/mL) and low Galectin‐9 group (<2038 pg/mL) according to the Youden index. The Kaplan–Meier curve results showed that the high Galectin‐9 group had a poor prognosis (*χ* ^2^ = 8.79, *p* < .05; Figure 1E).

Aside from clinical staging, previous studies have identified CD38, ZAP‐70, cytogenetic abnormality, serum β2‐microglobulin (β2‐MG), and serum lactate dehydrogenase (LDH) as prognostic factors for CLL.^1^, ^17^ Based on the expression of CD38, 53 CLL patients were divided into a CD38^+^ group (≥30%, *n* = 19) and a CD38^−^group (<30%, *n* = 34). Our findings showed that soluble Galectin‐9 was significantly higher in the CD38^+^ group than that in CD38^−^ group (*p* <.01). Among the 53 CLL patients, 23 were found to be ZAP‐70 positive (≥20%). Our data showed that soluble Galectin‐9 was significantly higher in the ZAP‐70^+^ group compared to the Zap‐70^−^ group (*p* < .01). Next, we defined CLL patients with normal karyotypes and del13q as a fine prognosis‐associated chromosome group (*n* = 32), whereas those with trisomy 12, del11q, del17p, and complex abnormal karyotypes were classified into a poor prognosis‐associated chromosome group (*n* = 21). We found that soluble Galectin‐9 was significantly higher in the poor prognosis‐associated chromosome group compared to the fine prognosis‐associated chromosome group (*p* < .01). Furthermore, based on their serum β2‐MG test results, CLL patients were divided into a β2‐MG < 3.5 mg/L group (*n* = 35) and a β2‐MG ≥ 3.5 mg/L group (*n* = 18). Soluble Galectin‐9 was significantly higher in patients in the β2‐MG ≥ 3.5 mg/L group compared to patients in the β2‐MG < 3.5 mg/L group (*p* < .01). Moreover, according to their serum LDH test results, CLL patients were divided into an LDH < 250 U/L group (*n* = 43) and an LDH ≥ 250 U/L group (*n* = 10). There were no significant differences in soluble Galectin‐9 between the two groups (*p* > .05; Table 2).

**Table 2:** Levels of Galectin‐9 in different prognostic groups of CLL patients ($\bar{x}\pm s$).

Index		Galectin‐9 (cases)	*t*	*p*
CD38			3.95	<.01
<30%		34		
≥30%		19		
ZAP‐70			4.67	<.01
<20%		30		
≥20%		23		
Chromosome			2.5	<.05
Fine		32		
Poor		21		
β2‐MG (mg/L)			2.35	<.05
<3.5		35		
≥3.5		18		
LDH (U/L)			0.34	>.05
<250		43		
≥250		10		

Abbreviations: β2‐MG, β2‐microglobulin; CLL, chronic lymphocytic leukemia; LDH, lactate dehydrogenase.

### 3.3 Tim‐3 overexpressed in CLL patients

**Figure 2:**
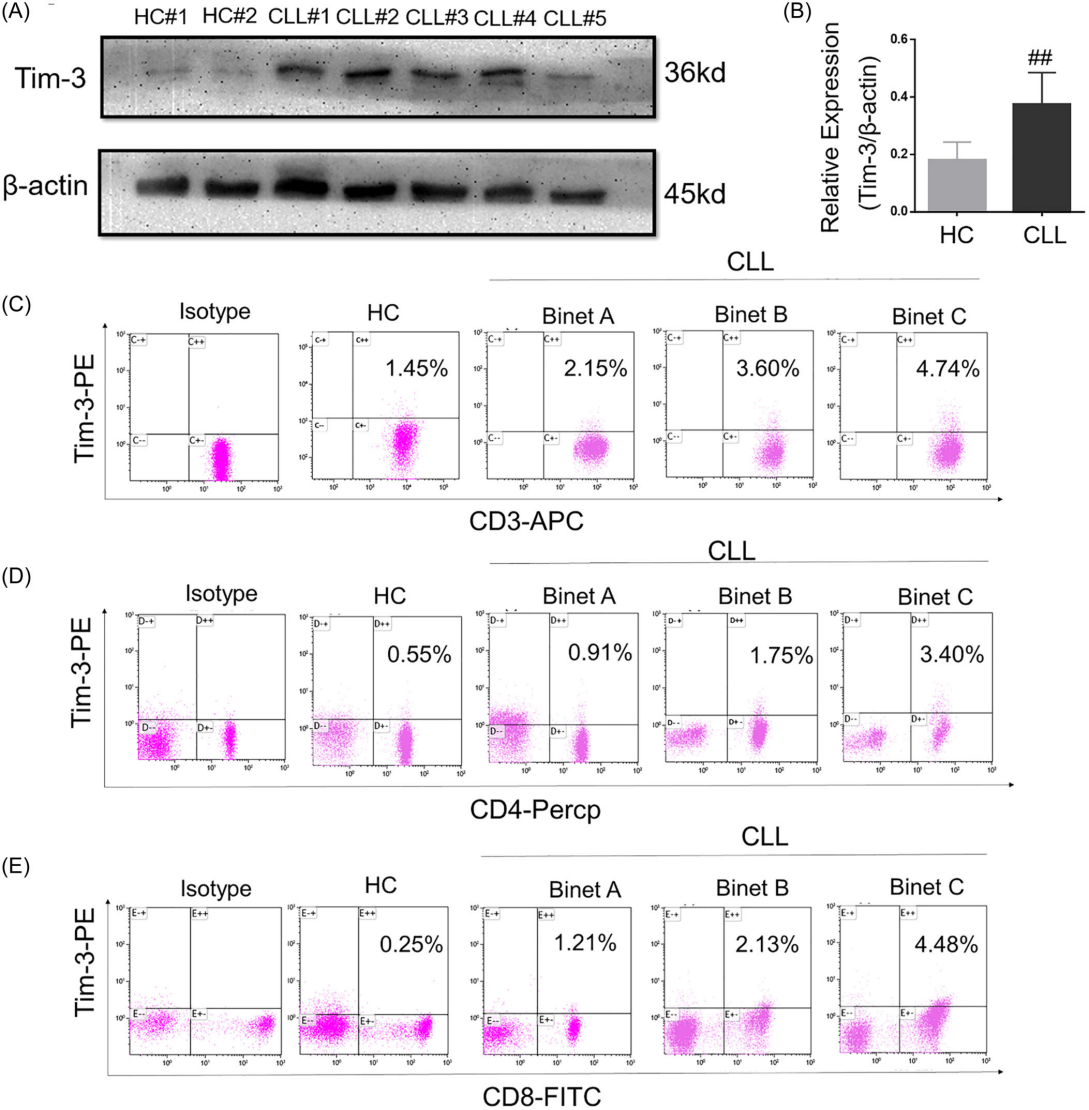
Tim‐3 expression in CLL patients. (A, B) Tim‐3 protein expression in HC and CLL groups. (C–E) Tim‐3 expression on the surface of T (CD3^+^, CD4^+^, and CD8^+^) cells in HC and CLL groups, as well as different Binet stages of CLL patients. Compared with the HC group: ^##^ *p* < .01, *p < .05, **p < .01. HC, healthy control, CLL, chronic lymphocytic leukemia.

We observed the expression of Tim‐3 protein in peripheral PBMC from five patients with CLL and two healthy controls (Figure 2A). The CLL group showed a significant increase in the Tim‐3 level compared to the HC group (Figure 2B). We detected Tim‐3 expression on T cells (CD3^+^T cells, CD4^+^T cells, CD8^+^T cells) by flow cytometry, finding that Tim‐3 expression on CD3^+^T cells, CD4^+^T cells, and CD8^+^T cells in patients in the CLL group was relatively higher than that in HC group. Furthermore, Tim‐3 expression on CD3^+^T cells, CD4^+^T cells, and CD8^+^T cells was significantly different among the three Binet stage groups of CLL patients; the Tim‐3 expression on T cells became more abundant in the later Binet stage in the CLL group (*p* < .05, *p* < .01; Figure 2C–E and Table 3).

**Table 3:** Tim‐3 expression on the surface of T cells in HC and CLL groups and different Binet stages of the CLL group.

Index	HC group	CLL group	CLL group		
			Binet A	Binet B	Binet C
Tim‐3^+^CD3^+^T cells (%)	1.45 ± 0.47	3.39 ± 1.44^a^	2.14 ± 0.5	3.65 ± 1.75^b^	4.74 ± 0.76^b^ ^,^ ^c^
Tim‐3^+^CD4^+^T cells (%)	0.55 ± 0.26	1.91 ± 1.04^a^	1.01 ± 0.35	1.86 ± 0.7^b^	3.41 ± 0.74^b^ ^,^ ^d^
Tim‐3^+^CD8^+^T cells (%)	0.38 ± 0.25	2.51 ± 1.43^a^	1.19 ± 0.48	3.41 ± 0.74^b^	4.61 ± 0.82^b^ ^,^ ^d^

Abbreviations: CLL, chronic lymphocytic leukemia; HC, healthy control.

^a^ *p* < .01 versus HC group.

^b^ *p* < .01 versus Binet A.

^c^ *p* < .05 versus Binet B.

^d^ *p* < .01 versus Binet B.

### 3.4 MDSCs increased in CLL patients

**Figure 3:**
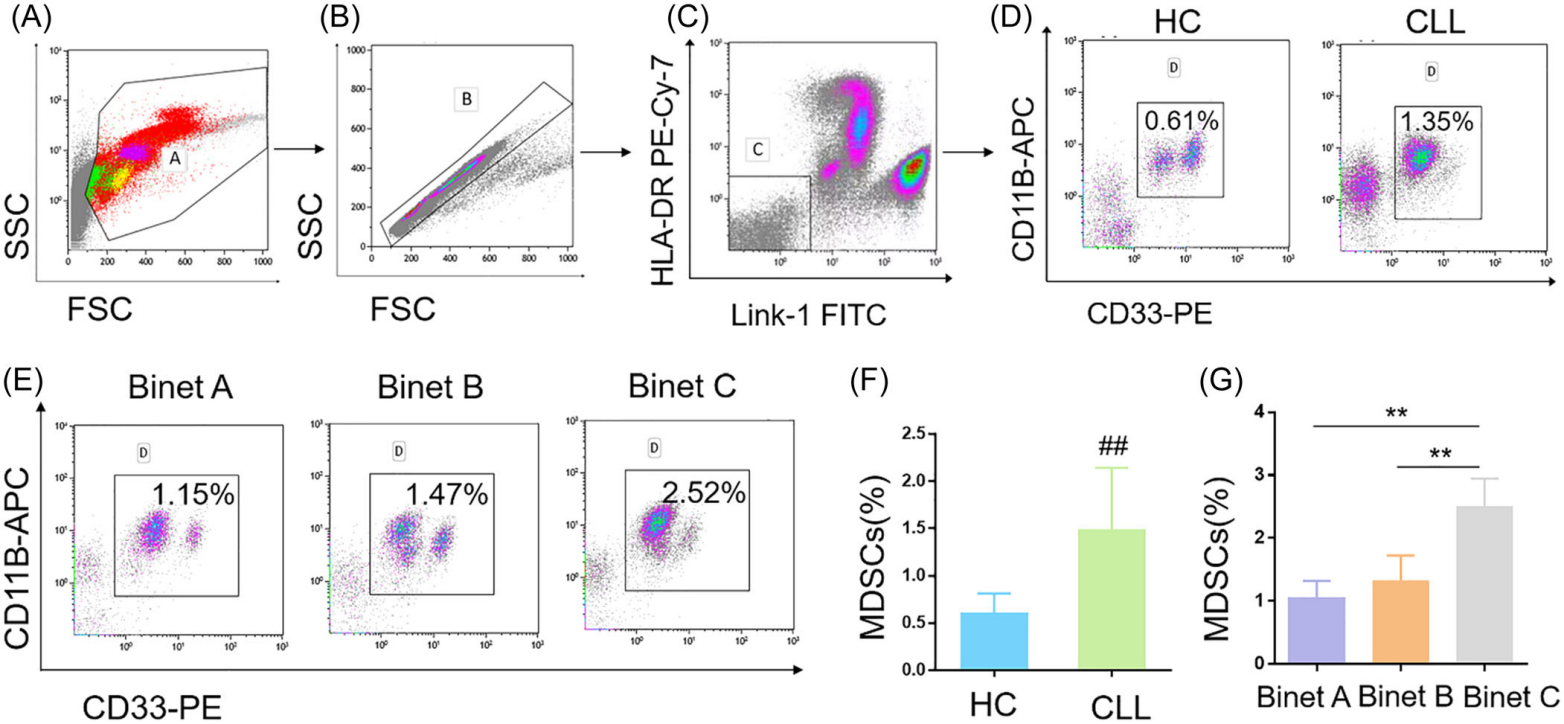
Changes of MDSCs in CLL patients. (A–C) Cells were first gated from FSC/SSC, then HLA‐DR^−^Lin‐1^‐^ cells were detected. (D, F) To analyze MDSC in CD11b and CD33. Frequency of MDSCs in HC and CLL groups. (E, G) Frequency of MDSCs in CLL patients of different Binet stages. The first picture of FSC/SSC flow plots showing the gating strategy used to identified MDSCs (A, B). Compared with the HC group: ^##^ *p* < .01. **p < .01. CLL, chronic lymphocytic leukemia; HC, healthy control; MDSC, myeloid‐derived suppressor cell.

We detected the MDSCs in the peripheral blood of CLL and HC groups by flow cytometry. Cells were first gated from FSC/SSC, then HLA‐DR^−^Lin‐1^−^ cells were selected (Figure 3A–C). MDSCs were identified as CD33^+^CD11b^+^ cells. Our results showed that the frequency of MDSCs in the CLL group was significantly higher than that in the HC group (*p* < .01; Figure 3D,F). We further analyzed the changes of MDSCs in the Binet stage, finding that the frequency of MDSCs in the Binet C stage was higher than that in Binet A stage and Binet B stage (all *p* < .01); however, there was no difference between Binet A stage and Binet B stage (*p* > .05; Figure 3E,G).

**Figure 4:**
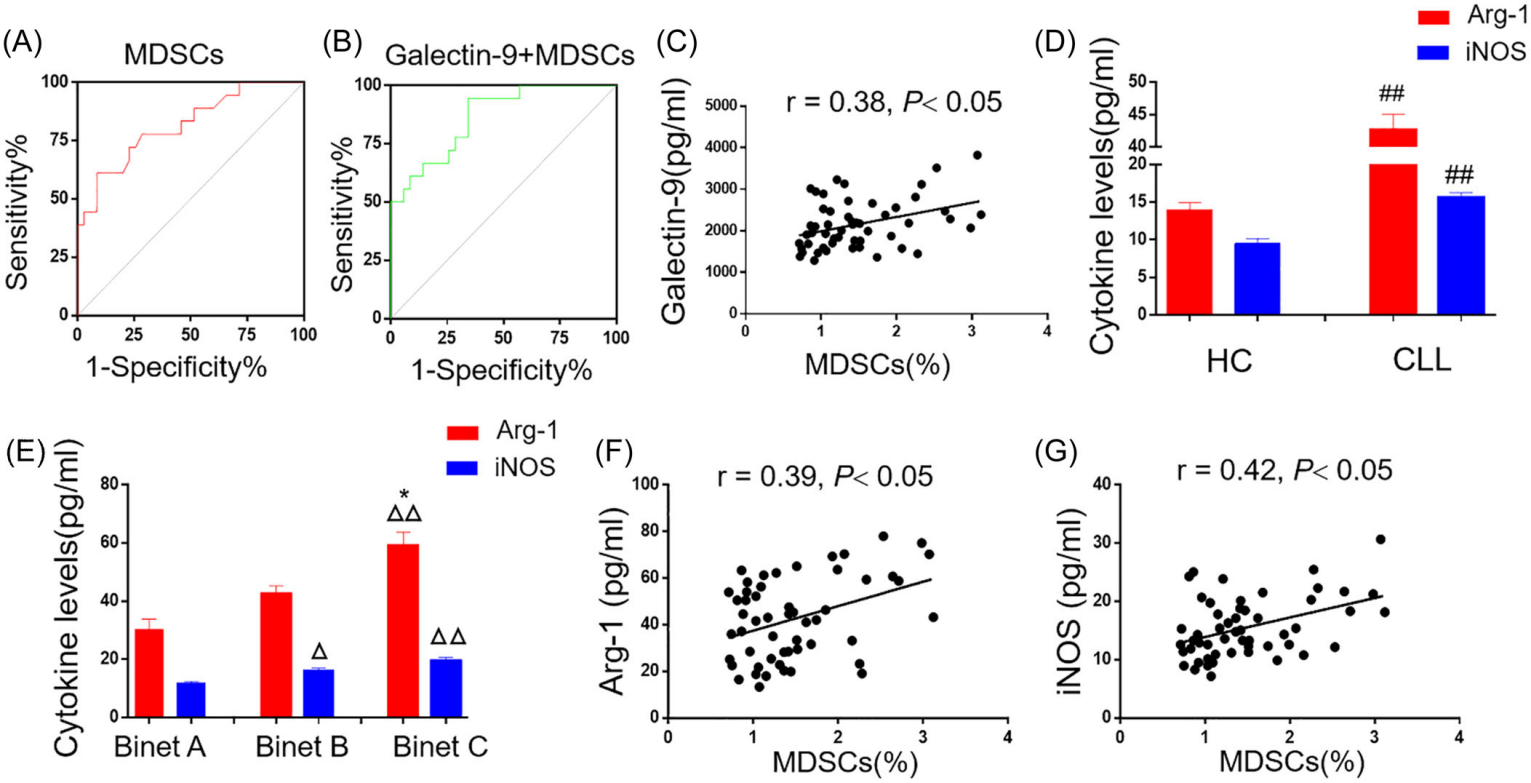
ROC cure and correlation analysis. (A) ROC curve of MDSCs based on the CLL patients of early stages and advanced stages (AUC = 0.816; 95% CI = 0.692–0.939; *p* < .05; sensitivity = 61.11%; specificity = 91.43%). (B) Combination of Galectin‐9 and MDSCs based on the CLL patients of early and advanced stages (AUC = 0.865; 95% CI: 0.716–0.964; *p* < .05; sensitivity = 94.44%; specificity = 65.71%). (C) Correlation between Galectin‐9 and MDSCs. (D) Levels of Arg‐1 and iNOS in HC and CLL groups. (E) Levels of Arg‐1 and iNOS in CLL patients of different Binet stages. (F) Correlation between Arg‐1 and MDSCs (*r* = .39, *p* < .05). (G) Correlation between iNOS and MDSCs (*r* = .42, *p* < .05). Compared with the HC group, ^##^ *p* < .01. Compared with Binet A group: ^△^ *p* < .05, ^△△^ *p* < .01. Compared with Binet B group: *p < .05. Arg‐1, argininase‐1; AUC, areas under the curve; CI, confidence interval; CLL, chronic lymphocytic leukemia; HC, healthy control; iNOS, inducible nitric oxide synthase; MDSC, myeloid‐derived suppressor cell; ROC, receiver operating characteristic.

Next, we analyzed CLL patients with the early and advanced stage to evaluate the prognostic value of MDSCs. ROC curve showed that sensitivity for MDSCs was 61.11% and the specificity was 91.43%. Youden index was 1.8%. (AUC = 0.816; 95% CI: 0.692–0.939; *p* < .05; Figure 4A). The AUC was increased to 0.865 when we combined the Galectin‐9 and MDSCs (sensitivity = 94.44%; specificity = 65.71%; 95% CI: 0.716–0.964; *p* < .05; Figure 4B). These results suggested that the combination of Galectin‐9 and MDSCs had good value in predicting CLL patients' prognoses compared with Galectin‐9 or MDSCs alone.

We further analyzed the correlation between soluble Galectin‐9 and MDSCs finding that soluble Galectin‐9 was positively correlated with MDSCs (*r* = .38, *p* < .05; Figure 4C).

### 3.5 The levels of Arg‐1 and iNOS increased in CLL patients

Our results revealed that the concentration of Arg‐1 and iNOS in the serum of the CLL group were significantly higher than that of the HC group by ELISA (*p* < .01; Figure 4D). The concentration of Arg‐1 in the Binet C stage was higher than that in the Binet A stage (*p* < .01) and Binet B stage (*p* < .05), but there was no significant difference between Binet A stage and Binet B stage (*p* > .05). The concentration of iNOS in the Binet B stage was higher than that in Binet A stage (*p* < .05). Binet C stage was higher than Binet A stage (*p* < .01); however, there was no significant difference between the Binet B stage and the Binet C stage (*p* > .05; (Figure 4E). Correlation analysis of MDSCs with Arg‐1 and iNOS showed that MDSCs were positively correlated with Arg‐1 and iNOS respectively (*r* = .39, *p* < .05; *r* = .42, *p* < .05; Figure 4F,G).

### 3.6 Univariable and multivariate Cox regression analysis of PFS in CLL patients

Age, sex, Binet stage, CD38, ZAP‐70, chromosome, β2‐MG, Galectin‐9, as well as MDSCs were included in univariate Cox regression analysis of PFS, and the results showed that Binet C stage (*p* < .01), CD38 ≥ 30% (*p* < .05), ZAP‐70 ≥ 20% (*p* < .01), β2‐MG ≥ 3.5(mg/L) (*p* < .05), Galectin‐9 ≥ 2039(pg/mL) (*p* < .01) and MDSCs ≥ 1.8(%) (*p* < .01) had adverse effects on PFS. Further multivariate Cox regression analysis of the above indicators showed that CD38 ≥ 30% (*p* < .01) and MDSCs ≥ 1.8(%) (*p* < .01) were independent adverse prognostic factors of PFS (Table 4).

**Table 4:** Univariable and multivariate Cox regression analysis of PFS in CLL patients.

Characteristic	Univariate		Multivariate analysis	
	HR (95% CI)	*p*	HR (95% CI)	*p*
Age ≥60	1.73 (0.74–5.20)	.19		
Male	1.42 (0.61–3.39)	.42		
Binet C stage	4.55 (3.68–36.52)	**<.01**	–	.20
CD38 ≥ 30%	2.79 (1.37–9.12)	**<.05**	0.22 (0.08–0.58)	**<.01**
ZAP‐70 ≥ 20%	3.24 (1.47–8.57)	**<.01**	–	.85
Poor chromosome	1.33 (0.55–3.43)	.51		
β2‐MG ≥ 3.5 (mg/L)	2.39 (1.06–6.07)	**<.05**	–	.25
Galectin‐9 ≥ 2039 (pg/mL)	3.53 (1.66–8.95)	**<.01**	–	.21
MDSCs ≥ 1.8 (%)	4.57 (3.57–31.19)	**<.01**	0.11 (0.04–0.33)	**<.01**

Abbreviations: β2‐MG, β2‐microglobulin; CI, confidence interval; CLL, chronic lymphocytic leukemia; HR, hazard ratio; PFS, progression‐free survival.

## 4 DISCUSSION

A variety of immune cells and negative signaling molecules participate in CLL development. Galectin‐9 possesses immunosuppressive effects^7^, ^17^; however, its clinical value in CLL has not yet been fully understood. We found that Galectin‐9 levels in CLL patients were significantly higher than that in healthy controls and increased with the progression of the Binet stage, which was consistent with the results of Wdowiak et al.'s^18^ study. In addition, ROC analysis results of this study showed that soluble Galectin‐9 had relatively high sensitivity and specificity in evaluating the disease progression of CLL. Meanwhile, we conducted PFS analysis on Galectin‐9 according to the cut‐off value obtained from the ROC curve, and we found that patients with high‐level Galectin‐9 had a poor prognosis. Previous studies showed that CD38, ZAP‐70, cytogenetic abnormalities, serum β2‐MG, and serum LDH are prognostic factors of CLL.^19^, ^20^, ^21^ To evaluate the potential prognostic value of Galectin‐9 in CLL, we analyzed the correlation between soluble Galectin‐9 and above indicators. We found that the concentration of soluble Galectin‐9 markedly increased in the CD38^+^ group, ZAP‐70^+^ group, poor prognostic chromosome group, and β2‐MG ≥ 3.5 mg/L group. Besides, univariate Cox analysis showed that Galectin‐9 was a risk factor for PFS in CLL patients. According to previous studies, the high concentration of Galectin‐9 in serum correlates with a poor prognosis in solid tumors, such as pancreatic and kidney cancer.^22^, ^23^ Another study found that Galectin‐9 levels in serum of patients with cutaneous T‐cell lymphoma are correlated with disease severity.^24^ Thus, we suggest that Galectin‐9 may be used as a new prognostic indicator of CLL patients.

Tim‐3 was found to be correlated with an immune escape both in solid tumors and hematological malignant tumors.^25^, ^26^ Galectin‐9/Tim‐3 signal pathway suppresses T cells, leading to tumors' occurrence and development.^27^ According to our previous studies,^4^, ^28^ the binding of Tim‐3 on Treg and Galectin‐9 promotes the function of Treg cells while inhibiting Th1 cells and CD4^+^T cells, which can be restored by blocking the Galectin‐9/Tim‐3 pathway in vitro experiments of CLL. In this study, we found that Tim‐3 was significantly increased in PBMC of CLL patients, and the percentage of Tim‐3 on T cells increased as the disease progressed. Consequently, we considered that Tim‐3 also participates in the disease development of CLL.

MDSCs are closely correlated with the development of solid tumors,^29^, ^30^ whose importance in hematological malignant tumors has not been appreciated until recently. Compared with the previously reported study results,^31^, ^32^ the ROC analysis of this study showed that MDSCs possess relatively high sensitivity and specificity for evaluating the disease progression of CLL. In addition, when combining Galectin‐9 with MDSCs for drawing ROC curves, the AUC was markedly improved. The results above suggest that the value of combined Galectin‐9 and MDSCs for evaluating the disease progression of CLL is higher than that of a single biomarker. To the best of our knowledge, this is the first study that demonstrated the value of combined Galectin‐9 and MDSCs for evaluating the disease progression of CLL patients. In addition, univariate and multivariate Cox regression analysis showed that MDSCs was an independent prognostic factor for PFS in CLL patients, which is an important newness of the current study. Galectin‐9 combined with MDSCs can help to predict the prognosis and guide the treatment of CLL patients.

Arg‐1 and iNOS produced by MDSCs are considered key factors inhibiting T cells.^33^ We found that the concentrations of Arg‐1 and iNOS in serum of CLL patients notably increased, which is correlated with the disease development. Further correlation analysis showed that Arg‐1 and iNOS are positively correlated with MDSCs. MDSCs possess immunosuppressive and protumorigenic capabilities in solid and hematological malignancies.^13^, ^34^ In addition, some scholars recently found that the upregulated Galectin‐9 in nasopharynx cancer affects the function of MDSCs.^35^ Yet, there are no relevant reports on whether Galectin‐9 affects MDSCs or participates in the CLL pathogenetic process. Our further analysis showed that Galectin‐9 was positively correlated with MDSCs, so we speculated that the elevated Galectin‐9 in CLL patients may affect the function of MDSCs. We think that the specific mechanism through which Galectin‐9 affects the function of MDSCs, thus participating in the pathogenesis of CLL, is worthy of further investigation. We hope that Galectin‐9 and MDSCs could be used as potential biomarkers and therapeutic targets for CLL in the future.

The present study has some limitations, First, we did not explore the mechanism of action and effect of Galectin‐9 on MDSCs through in vitro experiments. In addition, this study has a small sample size and a short follow‐up time. All these limitations should be taken into account in further studies. More research is needed to further confirm the prognostic value and specific mechanism of Galectin‐9 and MDSCs in CLL patients.

In conclusion, our data indicate that upregulation of Galectin‐9 and MDSCs were associated with clinical progression and worse prognosis of CLL patients. When used in combination, Galectin‐9 and MDSCs can become more meaningful biomarkers for guiding clinical practice. Thus, Galectin‐9 and MDSCs could be important prognostic indicators for CLL patients.

## AUTHOR CONTRIBUTIONS

**Xierenguli Alimu**: Formal analysis; investigation; writing—original draft; writing—review and editing. **Juan Zhang**: Formal analysis; investigation. **Nannan Pang**: Formal analysis; investigation. **Rui Zhang**: Formal analysis; investigation. **Rong Chen**: Data curation; validation. **Xuejiao Zeng**: Data curation; validation. **Shabaaiti Tudahong**: Data curation; validation. **Gang Chen**: Resources. **Maliya Muhashi**: Resources. **Fang Zhao**: Resources. **Jianbing Ding**: Conceptualization. **Jianhua Qu**: Funding acquisition.

## CONFLICT OF INTEREST STATEMENT

The authors declare no conflict of interest.

## Data Availability

The data that support the findings of this study are available from the corresponding author upon reasonable request.
